# Impaired Binaural Hearing in Adults: A Selected Review of the Literature

**DOI:** 10.3389/fnins.2021.610957

**Published:** 2021-03-19

**Authors:** Frederick J. Gallun

**Affiliations:** Oregon Hearing Research Center, Oregon Health and Science University, Portland, OR, United States

**Keywords:** lateralization, localization, binaural, spatial hearing, impairment, auditory

## Abstract

Despite over 100 years of study, there are still many fundamental questions about binaural hearing that remain unanswered, including how impairments of binaural function are related to the mechanisms of binaural hearing. This review focuses on a number of studies that are fundamental to understanding what is known about the effects of peripheral hearing loss, aging, traumatic brain injury, strokes, brain tumors, and multiple sclerosis (MS) on binaural function. The literature reviewed makes clear that while each of these conditions has the potential to impair the binaural system, the specific abilities of a given patient cannot be known without performing multiple behavioral and/or neurophysiological measurements of binaural sensitivity. Future work in this area has the potential to bring awareness of binaural dysfunction to patients and clinicians as well as a deeper understanding of the mechanisms of binaural hearing, but it will require the integration of clinical research with animal and computational modeling approaches.

## Introduction

The ability to process the information available in pressure waves arriving at the two ears (“binaural hearing”) is available to living creatures ranging from insects (Hedwig and Stumpner, 2016) to humans (for review see Stecker and Gallun, 2012). Binaural hearing has obvious defensive and predatory advantages, as well as serving an important communicative function. Consequently, dysfunction of the binaural system can reduce the ability to navigate the auditory scene (Gallun and Best, 2020). This review of binaural impairment in adult human listeners will start with an overview of the history of the area and the current model of the binaural system. After surveying a variety of methods of characterizing binaural impairment, the literature on patient groups will be selectively reviewed. This review of the patient literature will be divided into two main sections. The first will focus on patients with conductive hearing loss (CHL) and sensorineural hearing loss (SNHL). The second will turn to those patients with central dysfunction, for whom detection of pure tones is often normal or near normal but for whom binaural sensitivity has been shown to be impaired. The final section will address the many opportunities for additional studies that are made clear by what this review is and is not able to tell us about the mechanisms of binaural impairment and about the abilities of various patient groups to make use of the auditory spatial cues available in the environment.

Binaural function has been studied clinically from as far back as 1876 (Pierce, 1901). The importance of studying abnormal auditory function has been known from the very first studies of binaural hearing. The work of Venturi (1796) was described by Wade and Deutsch (2008) and Stecker and Gallun (2012). Venturi hypothesized, based on his comparisons of monaural and binaural listening, that the relative intensities at the two ears (the “interaural level difference”; ILD), give rise to the ability to localize sounds in space. Rayleigh (1907) was the first to go beyond the ILD and show that differences in the time of arrival of a sound at the two ears (the “interaural time difference”; ITD) is also a potent binaural cue for localization of sound sources. Even before the neural sites of binaural interaction had been identified, clinician scientists such as Greene (1929) and Walsh (1957) were applying psychoacoustical techniques to study the binaural abilities of their patients and using those results to form hypotheses about the underlying anatomy and physiology.

**FIGURE 1:**
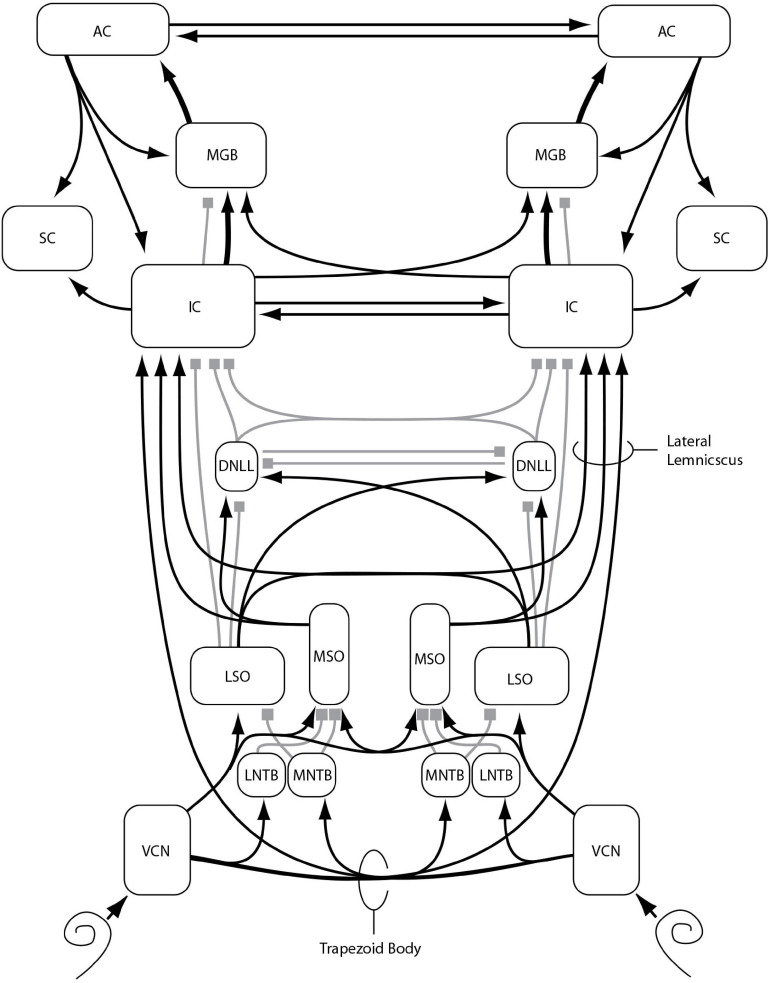
Major nuclei (boxes) and primarily excitatory (lines with arrows) or inhibitory (lines with squares) interconnections of the ascending auditory pathway, with an emphasis on binaural function and connectivity, as described in the text: VCN: ventral cochlear nucleus, MNTB: medial nucleus of the trapezoid body, LNTB: lateral nucleus of the trapezoid body, MSO: medial nucleus of the superior olivary complex, LSO: lateral nucleus of the superior olivary complex, DNLL: dorsal nucleus of the lateral lemniscus, IC: inferior colliculus, SC: superior colliculus, MGB: medial geniculate body of the thalamus, AC: auditory cortex. Additional nuclei, projections, and subdivisions are omitted for clarity. Reproduced from Stecker and Gallun (2012), in Translational Perspectives in Auditory Neuroscience: Normal Aspects of Hearing (p. 395) by Tremblay, K., & Burkard, R. Copyright^©^ 2012 Plural Publishing, Inc. All rights reserved. Used with permission.

Since these early clinical studies, much has been revealed about the anatomy and physiology of the binaural system. Figure 1 provides a schematic diagram of some of the main aspects of what Stecker and Gallun (2012) determined to be the currently accepted model of how the binaural system is connected physiologically, with an emphasis on the ascending binaural system. For further details of the basic architecture of this system see Yin (2002). Essential to the functioning of these pathways is high-fidelity transduction from the outer ear to the lateral and medial nuclei of the superior olivary complex (LSO; MSO) via the medial and lateral nuclei of the trapezoidal body (MNTB; LNTB). The reason for this is that we now know that both nuclei depend on microsecond (μs) temporal precision for the comparison of neural impulses from the two ears. As described in detail in Stecker and Gallun (2012), the discharge rates of LSO and MSO neurons can vary across nearly their entire response range when presented with only 1 millisecond (ms) of delay between the left and right ear inputs. Such precision, which is essential for allowing the system to be sensitive to ITDs as low as 10 μs (see Stecker and Gallun, 2012, Table 14-1) depends critically on the transformation of instantaneous pressure levels at the eardrum to spikes on the auditory nerve (Carr and Konishi, 1990). This transformation, which is known as neural phase-locking, allows the system to encode both the temporal fine structure (TFS) and the temporal envelope structure (TES) of the stimuli at each ear (Dreyer and Delgutte, 2006), from which LSO and MSO are able to accurately extract ILD and ITD information. For this reason, diseases that degrade neural transduction, such as multiple sclerosis (MS), are particularly likely to result in binaural impairment even when pure-tone thresholds are within the normal range.

Degradation of the transduction of pressure waves in the air to fluid motion in the cochlea, known as “conductive hearing loss” (CHL), can arise from blockages in the ear canal, perforations of the tympanic membrane, blockages of the middle ear, or dysfunction of the bones of the middle ear (the “ossicles”) due to breakage or stiffening. All of these could delay or distort neural phase-locking and introduce either systematic or random error into the binaural analysis, especially if there was a substantial asymmetry between the two ears. The next site of potential dysfunction is the cochlea itself, impairment of which is known as SNHL. SNHL is defined as involving the basilar membrane, the fluids of the cochlear ducts, the organ of Corti, inner, and outer hair cells, and the stria vascularis, which is responsible for the blood supply to the cochlea. Damage or dysfunction at any of these sites has the potential to reduce both the amount of neural signaling as well as the accuracy of phase-locking to the pressure waves reaching the outer ear.

While the clinical diagnosis of CHL and SNHL relies upon the detection of pure tones presented via earphones (air-conduction, AC) or bone-vibration (bone-conduction, BC), it does not involve any measure of phase-locking. If AC and BC measurements reveal similar thresholds, but detection requires higher tone levels than is normal for a tone of that frequency, as specified by international standards (e.g., ANSI/ASA S3.6, 2018), then CHL is ruled out and SNHL is suspected. This renders diagnosis of SNHL and CHL insufficiently precise for assessing the potential impact on the binaural system. All impairment beyond the cochlea, including dysfunction of the auditory nerve, is known as retrocochlear hearing loss (RHL), the modern diagnosis of which depends largely on imaging, although it can also involve the auditory brainstem response (ABR), which assesses neural timing. The vast majority of RHL diagnoses are due to vestibular schwannoma (also called acoustic neuroma), which is a benign tumor that grows on the vestibular portion of the eighth nerve. As it grows, this tumor damages the nerve and, if allowed to grow large enough, can damage the cochlear nucleus (CN) and other brainstem structures. For further details on the causes, diagnosis, and categorization of the types and severity of hearing losses, see Katz (2014).

As can be seen in Figure 1, there are multiple sites along the neural auditory pathway where damage or disease could result in binaural impairment. The first is on the auditory nerve itself, where signal transduction and transmission can be reduced by loss or dysfunction of synapses and/or auditory nerve fibers. Even if the signals arriving at the synapse of the auditory nerve with the CN are not degraded or reduced, there is the possibility of dysfunction within the CN or in the signaling pathway to the trapezoidal body, either due to damage to those pathways or demyelination, which could impede or delay neural impulses and introduce random errors into binaural comparisons. In addition to the trapezoidal body and the superior olivary complex, the dorsal nucleus of the lateral lemniscus (DNLL), the inferior colliculus (IC), the medial geniculate body of the thalamus (MGB), and auditory cortex (AC) all are involved in conveying and processing binaural information. As such, damage or delays at any of these sites could degrade the transformation of binaural information into spatial maps and the ability to assign spatial locations to perceived objects.

## Methods of Characterizing Binaural Impairment

**FIGURE 2:**
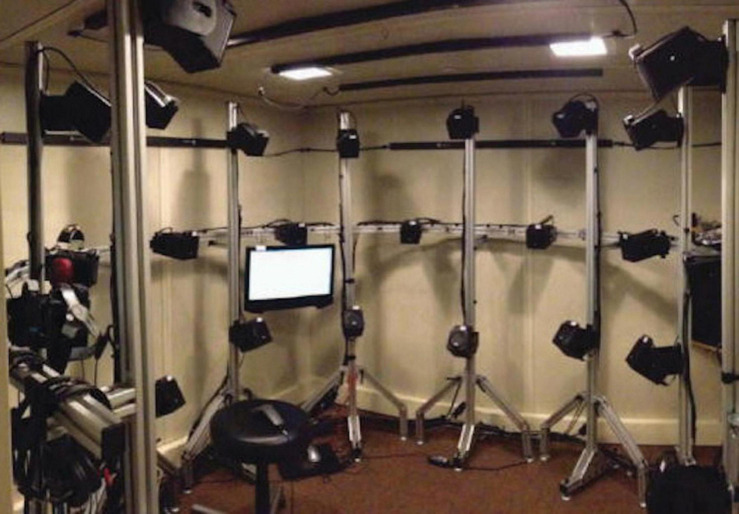
Example of a loudspeaker array used to test localization ability by identification of the loudspeaker from which a test signal has been presented. Such arrays can also be used to test spatial release from masking with speech or other stimuli. See text for experimental details. Reproduced with permission from Brungart et al. (2017). Copyright 2017, Acoustical Society of America.

All of the earliest work on impaired localization (reviewed in Pierce, 1901) appears to have relied upon either anecdotal reports or examinations of the ability to identify the location of sounds in a test room. One of the earliest methods of quantifying localization ability beyond simply determining whether it was accurate or inaccurate, was that of Jongkees and Van der Veer (1957), who presented sounds from one of eighteen loudspeakers and participants pointed to the perceived location with their eyes closed. Response deviations were compared to those of a group of participants with no known peripheral or central pathology. Based on whether the deviations were within this range or not, the patients were categorized as having “normal” or “pathological” localization. Häusler et al. (1983) conducted the first localization experiments using discrimination tasks, in which two intervals are presented, only one of which contains a target. Discrimination is preferable to methods of adjustment or identification because it allows sensitivity to a stimulus to be measured independently of the expectations or willingness of the observer to make a particular response (Green and Swets, 1974). Using a loudspeaker array in an anechoic chamber, a two-interval forced-choice procedure was used to test the minimum audible angle (MAA; Mills, 1958) in the horizontal and vertical planes. Akeroyd and Whitmer (2016) reviewed 29 studies that used a variety of localization tasks, most of which involved pointing or identifying source locations rather than discrimination. Both types of tasks can be done either with real speakers (either in an array with fixed locations or on a movable boom) or using a virtual acoustical simulation (VAS; Brungart et al., 2017). To create a VAS, head-related transfer functions (HRTFs) are imposed on the test stimuli, allowing the acoustic cues associated with a given spatial location of the stimulus to be presented over headphones (Wightman and Kistler, 1989). An example of a speaker array that was used for localization experiments with a closed set of fixed loudspeakers is shown in Figure 2.

In addition to tests of localization, it is also desirable to separate out the various acoustical cues and test sensitivity to each independently. The first tests of binaural sensitivity to ITD and ILD were conducted by Greene (1929), using a custom-made device that allowed ITD to be manipulated by changing the length of a column of water through which the sound was conducted, and thus the speed of travel through the fixed-length tube. ITD was not measurable with this device, but deviations from normal ITD sensitivity could be detected. Similarly, attenuation of the signal reaching one ear or the other allowed ILD to be manipulated in a manner that allowed abnormal sensitivity to be detected. Using headphones and modern electronics, Häusler et al. (1983) estimated the Just Noticeable Difference (JND) in ITD and ILD. The JNDs were measured with stimuli set to equal “sensation level” (SL), which is defined as a given level above detection threshold, thereby controlling for differences in hearing thresholds between ears. This basic approach has been used many times since (Hawkins and Wightman, 1980; Smoski and Trahiotis, 1986).

Another approach to measuring binaural ability, inspired by the work of Licklider (1948), is the binaural masking level difference (MLD). The MLD is defined as improved performance on a masked tone detection or speech identification task that is associated with the imposition of interaural differences on either the target or the masker. Melnick and Bilger (1965) conducted the first study of the MLD in patients, using a speech stimulus. Olsen et al. (1976) and Olsen and Noffsinger (1976), were the first to measure the MLD for a 500 Hz pure tone, which is more common in modern studies than is the use of speech targets.

A more recent approach to behavioral testing of binaural function involves spatial release from masking (SRM; Marrone et al., 2008), which is similar to the speech MLD but involves a loudspeaker array such as that shown in Figure 2, or a VAS. The first approaches (Duquesnoy, 1983; Bronkhorst and Plomp, 1989; Peissig and Kollmeier, 1997; Arbogast et al., 2005) presented a target sound from one location and a masking sound from another location, as well as varying the interfering sounds to include competing speech. However, these studies suffered from the confound that when the masker was presented at a single location, different signal to noise ratios (SNRs) were available at the two ears. This in turn leads to the availability of a “better-ear” listening strategy, which means that performance may improve even if the listener has no binaural sensitivity whatsoever. Using a method suggested by the manipulations and better-ear calculations of Hawley et al. (2004); Marrone et al. (2008) demonstrated that the better-ear effect can be eliminated (at least on a long-term basis) by displacing two maskers symmetrically to the left and right of the target. Gallun et al. (2013) introduced a VAS version of this test and a testing procedure based on the single descending track or “progressive tracking” method. This procedure is very fast (under 10 min) but is better suited for detecting abnormal performance than for obtaining precise measurements of threshold (Gallun et al., 2015). Ellinger et al. (2017) took advantage of the VAS to present processed speech signals in which ITDs and ILDs were manipulated independently and thus could be either reinforcing or conflicting, as Colburn and Durlach (1965) had done using an MLD paradigm.

**FIGURE 3:**
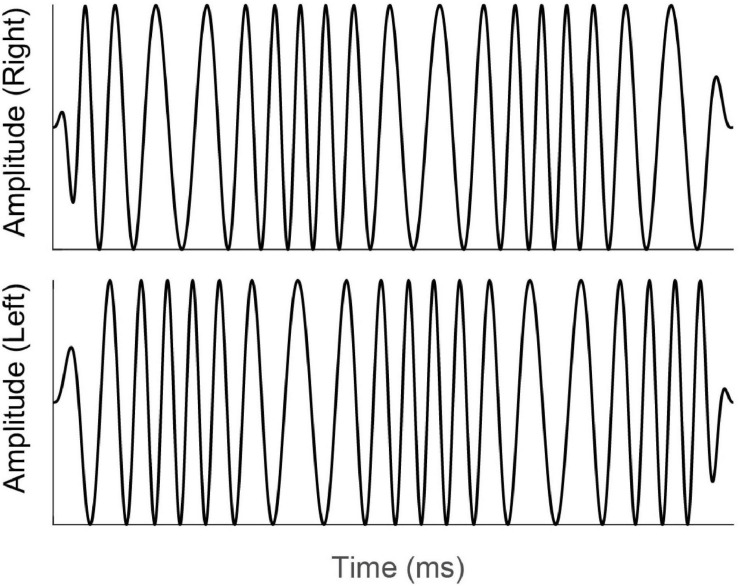
Schematic diagram of the time-amplitude waveforms of a dichotic FM stimulus. For illustration purposes, the carrier frequency has been reduced and modulation depth increased from the values that would be used experimentally. Note that while there is no onset or offset difference in amplitude or phase, the phases of the signals at the left and right ears (top and bottom waveforms) are continually changing, resulting in an interaural phase difference that changes over time and an interaural time difference that shifts from left-leading to right-leading and back again. Reproduced under Creative Commons reuse license from Koerner et al. (2020).

An additional method for testing binaural sensitivity that has gained popularity recently is similar to the MLD, in that it involves the detection of interaural phase differences (IPDs), but instead of detecting a signal in noise, the task asks the listener to report directly on their binaural percept. The earliest work (Green et al., 1976; Witton et al., 2000) involved presenting a static tone to one ear and a tone to which frequency modulation (FM) had been applied to the other ear. It was found that for people with normal hearing, the presence of FM in the target stimulus was detectable at lower modulation depth when the FM resulted in IPDs than when the same FM was presented monaurally. Grose and Mamo (2012) extended this paradigm by presenting FM diotically (same FM at both ears) or dichotically (FM reversed in phase at the two ears). An example of a dichotic FM stimulus is shown in Figure 3.

**FIGURE 4:**
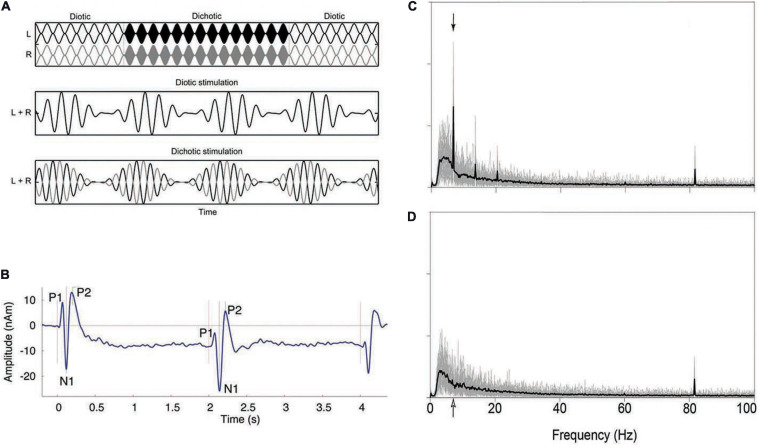
Example of a stimulus that shifts from diotic to dichotic **(A)** and the evoked response generated in the time domain (P1-N1-P2 complex; **B**) and frequency domain (IPM-FR; **C,D**). See text for further details. Note that the stimulus shown shifts from diotic to dichotic and back to diotic, while the stimulus that would be used to evoke the P1-N1-P2 complex shown in panel **(B)** would only shift once, from diotic to dichotic, at the temporal midpoint of the stimulus. The stimulus used to generate the IPM-FR would contain many such alternations, at a characteristic rate, usually between 5 and 10 Hz. The arrows in panels **(C,D)** indicate the frequency at which the stimulus used to generate the IPM-FR alternated from diotic to dichotic, which in this case was 6.8 Hz. Additional evoked responses shown in panel **(B)** indicate the onset and the offset of the signal, while those in panels **(C,D)** indicate the response to the amplitude modulation rate (81.6 Hz) of the 500 Hz carrier. Additional low-frequency peaks in panel **(C)** represent aliasing at integer multiples of the IPM rate of 6.8 Hz. Panel **(D)** shows the response to a diotic stimulus and thus does not contain the peaks indicating the presence of the IPM-FR but does show the response to the modulation of the carrier amplitude at 81.6 Hz. Reproduced with permission from Ross et al. (2007); Vercammen et al. (2018), and Koerner et al. (2020). Vercammen et al. copyright 2018, Sage Publications. Ross et al. copyright Journal of Neuroscience. Koerner et al. reused under Creative Commons license.

In addition to behavioral methods, researchers have also used neurophysiological responses from cortical neurons to compare binaural responses in patients to those found in control participants. That binaural signals produce different neurophysiological responses than do diotic signals has been known for many years (Butler and Kluskens, 1971; Fowler and Mikami, 1992), but only recently have these responses been measured in patient groups. Ross et al. (2007), using magnetoencephalography (MEG), measured the auditory evoked responses (P1-N1-P2 complex) in response to binaural changes in the middle of ongoing signals, and later researchers (Papesh et al., 2017; Eddins and Eddins, 2018) extended this to electroencephalography (EEG). The P1-N1-P2 complex, which is measured in the time domain, arises at multiple levels of the thalamus and AC (Haywood et al., 2015). More recently (Haywood et al., 2015; Anderson et al., 2018; Vercammen et al., 2018; Koerner et al., 2020) researchers have used sequences of stimuli that changed rapidly (<7 Hz) in binaural configuration and measured the interaural phase modulation following response (IPM-FR). These responses are believed to be generated from the same sites as the P1-N1-P2 complex, but the steady-state nature allows them to be examined in the frequency domain. The amplitude of the response is used to quantify the degree to which changes in neurophysiological responses are correlated with the changes in binaural configuration. Figure 4A shows the stimuli used to generate the IPM-FR, which is shown in Figure 4C. The stimuli shown in Figure 4A are also similar to those used to generate the P1-N1-P2 complex, which is shown in Figure 4B. Figure 4D shows the response to a diotic stimulus, which does not generate the IPM-FR. Thus, the comparison of Figures 4C,D shows how to identify the IPM-FR peak in Figure 4C. Further examples of the P1-N1-P2 complex can be seen in Figure 12.

## Studies on Binaural Function With Participants With Peripheral Hearing Loss

The section on methods of characterizing binaural impairment describes a range of techniques that have all been applied to listeners with peripheral hearing loss (CHL and/or SNHL). This section will review a selection of those studies, focusing on the earliest studies that pioneered the methods and then moving on to those studies with the largest number of participants and the most comprehensive methods. While tentative conclusions are drawn in some cases, the field of clinical research in the area of binaural dysfunction is still developing its evidence base. For this reason, the emphasis of this, as of the later sections, is as much on what has been done as on what has been learned.

### Peripheral Loss and Localization and Lateralization

Pierce (1901) describes a number of experiments using sophisticated equipment for testing the localization abilities of those with normal hearing, but only anecdotal evidence and simple localization tasks are described in the sections on people with impaired hearing. In one of the earliest descriptions of a localization experiment in people with hearing loss, Greene (1929) conducted experiments on eight patients with unilateral or bilateral CHL caused by otitis media. Using a “short-circuited binaural stethoscope” composed of section of rubber tubing connected to the two ear-pieces of a stethoscope, he measured how far from midline he had to tap a pencil on the tubing before patients could detect the displacement of the taps. All of these patients were able to detect an average displacement from midline of only 2.6 cm, which was not different from the detection thresholds of a normal-hearing control group.

Jongkees and Van der Veer (1957) tested 61 patients with hearing loss using the loudspeaker-based localization methods described in section “Methods of Characterizing Binaural Impairment.” Listeners were divided into seven groups based on etiology of hearing impairment [chronic otitis media, atresia, SNHL, otosclerosis (unoperated and operated), patients after “reconstructive radical mastoid surgery” or tympanoplasty, unilateral total deafness], and compared the results to the localization abilities of 40 people with normal hearing. Across multiple groups of patients, 72% of those patients tested (44/61) had “pathological” localization, while only 18% (11/61) reported difficulties when asked about localization in their daily lives. Otosclerosis, with or without surgery, and atresia were both associated with abnormal directional hearing in all patients, while in every other group at least a third of the patients had localization functions that were statistically indistinguishable from those in the normal hearing group.

Jongkees and Van der Veer (1957) also measured pure-tone detection thresholds by employing both air- and bone-conducted audiometry at 1,000 Hz, but found that this was not a very reliable indicator of who would be able to perform their localization task. As discussed by Durlach et al. (1981), there is no discussion of whether or not head movements were allowed, and duration of disease is not reported. Indeed, the discussion of the role of head movements as a cue to localization for people with unilateral deafness in Jongkees and Van der Veer (1958) suggests that this cue was available and may explain some of the variability in performance among the patients tested. So, it is possible that some of the patients with better localization had been living with the disease for years and had learned to localize using monaural cues and/or head movements, while some of the patients with worse localization were newly suffering and had not developed these skills.

The first tests of clinical patients’ binaural function using modern methods, in which sensitivity is dissociated from response bias, occurred in the 1980s. Prior to this time, all of the tests involved methods that were susceptible to response bias. For example, the localization of single sounds through identifying a location or adjusting knob can be influenced by expectations of where the sounds are likely to appear. Similarly, pressing a button when a sound is presented or repeating a spoken word depends on the willingness of the listener to report what they experienced. Discrimination tasks, in which two intervals are presented, only one of which contains a target, allows sensitivity to a stimulus to be measured independently of the expectations or willingness of the observer with regard to making a response (Green and Swets, 1974). Häusler et al. (1983) used discrimination tasks to measure both localization and sensitivity to interaural differences. For the localization task, which measured the MAA, or the ability to distinguish two loudspeaker locations, the participant was asked to discriminate two 1-s bursts of a broadband noise at a test location from two 1-s bursts presented at a reference location. Reference locations were either in front (“front-referenced MAA”) or to the side (“side-referenced MAA”), and test locations were either displaced horizontally or vertically. The experimenter varied the size of the differences adaptively, based on past performance, in an attempt to find the value that led to 80% correct performance. No feedback was given to the participant.

Häusler et al. (1983) reported data from 49 patients with peripheral hearing loss and 39 normal-hearing control listeners. Of the patients, 14 had bilateral SNHL, 17 had conductive losses, 9 had one deaf ear (no behavioral response to sound), and 9 had unilateral loss due to Ménière’s disease (for details on the audiological configurations associated with the disease, see Belinchon et al., 2011). A main finding of this study in terms of localization abilities of these patients was that, as had been found with earlier work, even in participants with similar etiologies, the ability to detect pure-tones was not a useful predictor of binaural function. It was revealed, however, that high-frequency hearing loss was associated with impairments in the ability to make vertical discriminations. In addition, the patients with SNHL and poor speech understanding were impaired on the vertical MAA and the side-referenced horizontal MAA, while the participants with similar audiograms and good speech understanding had good performance on all the tasks. The authors speculated that spectral discrimination deficits were probably responsible for the relationship between localization and speech discrimination, but as only broadband noise was tested, it was impossible to know how participants would have performed with narrowband stimuli.

Noble et al. (1994, 1997) also found only weak relationships between pure-tone detection ability and horizontal localization and replicated the finding that the lack of audibility associated with high-frequency hearing loss reduces access to spectral cues important for vertical localization and front-back discrimination. However, they were unable to replicate the relationship observed by Häusler et al. (1983) between localization and speech in noise ability. Abel et al. (2000) and Dobreva et al. (2011), although focused on the issue of aging, also reported that even relatively mild high-frequency hearing loss interferes with vertical localization. Neither study was able to clearly show how the detection of pure-tones or aging is related to horizontal localization, despite the fact that both lead to increases in between-subject variability.

To better understand the sources of between-subject variance in localization, Neher et al. (2011) tested the role of cognition and two measures of auditory processing ability (monaural spectral ripple discrimination and binaural TFS sensitivity) in a group of 23 older listeners (aged 60–78 years; mean of 67 years) with audiometric thresholds outside the normal range (pure tone average, “PTA” of 27–53 dB; mean of 41 dB HL) and a group of 8 younger listeners (aged 20–44 years; mean of 35 years; thresholds of 20 dB HL or better below 6 kHz). While the older listeners did more poorly than did the younger listeners on a loudspeaker identification task in an anechoic chamber with a speaker array with 15° horizontal separations between speakers, none of the other tests predicted performance. Neither age nor PTA was significantly correlated with localization performance.

Brungart et al. (2017) compared localization in anechoic and virtual conditions and found that those with hearing loss suffered an overall reduction in performance, which was associated both with increased age and pure-tone thresholds. Best et al. (2011) tested hearing-impaired listeners in both a quiet condition and in a condition with interfering sounds and found that while they performed similarly to normally hearing controls in quiet, performance was worse by about 7° in the presence of interference. Buchholz and Best (2020) followed up on this experiment using a more realistic listening environment and found that four of the fifteen subjects with hearing loss had particularly poor localization in quiet and in the presence of masking sounds, and that the worst performers were those with low-frequency hearing loss. This is an important caveat to the general finding of a lack of a relationship between pure-tone thresholds and localization, which should be specifically examined in future work.

**FIGURE 5:**
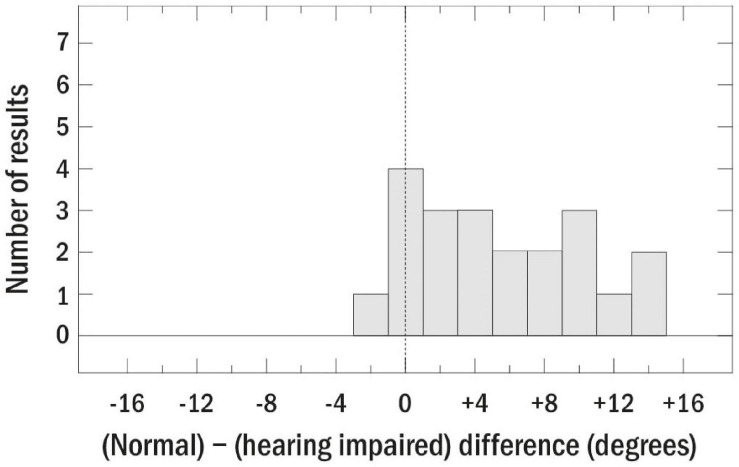
Summary plot of the differences between localization accuracy for normally hearing and hearing-impaired listeners for 29 studies review by Akeroyd and Whitmer (2016). The dashed line indicates similar acuity for those with and without peripheral impairment. Reproduced with permission from Akeroyd and Whitmer (2016). Copyright 2016, Springer Nature.

While this is only a sample of the data that have been collected on localization abilities of those with peripheral impairment, the interested reader is referred to Akeroyd and Whitmer (2016), who reviewed 29 studies of bilateral SNHL conducted between 1983 and 2014. Figure 5, which is reproduced from that chapter, summarizes the results of those studies by plotting the within-study differences in localization accuracy between the normally hearing and hearing impaired groups. Positive values, which represent the majority of the data, indicate worse acuity for those with peripheral impairment. Akeroyd and Whitmer (2016) concluded that, while the effects of age and hearing loss are difficult to separate, it seems likely that hearing loss results in about a 5° decrease in left–right localization accuracy. Furthermore, the size of the relationship between localization accuracy and hearing loss is fairly weak, amounting to a correlation of about 0.40.

Overall, the studies described above, as well as those reviewed by Akeroyd and Whitmer (2016), emphasize that while it is clear that hearing loss has a negative effect on localization, the audiogram is not sufficient for characterizing the mechanisms underlying the effect, at least for those with high-frequency hearing loss. Further work on these relationships, especially for those with low-frequency hearing loss and asymmetrical losses, is likely to be most productive when performed in combination with, or when informed by, animal and/or computational modeling. While low correlations and small effect sizes can be important for understanding the mechanisms and effects of peripheral hearing loss on localization ability, it is difficult to obtain significant results without running very large samples. Furthermore, the low correlations suggest that a predictive model of individual localization performance relevant for diagnosis and rehabilitation in a clinical setting will need to consider the influence of other factors such as age, cognition, and the integrity of the brainstem and auditory cortical pathways. For example, and as mentioned above, it is quite likely that for a patient with a particular hearing loss, localization ability may improve over time as they gain more awareness of localization cues and strategies that are specific to their binaural abilities. One way of getting more mechanistic insight into the ability of individual listeners to use spatial cues is to test sensitivity to ITD and ILD independently rather than through localization tasks.

### Peripheral Loss and Binaural Sensitivity to Interaural Differences

Häusler et al. (1983), in addition to measuring the MAA, measured the JND for ITDs and ILDs. For the interaural JND task, the participant was asked to discriminate two 1-s bursts of a broadband noise to which an ITD or ILD had been applied from two 1-s bursts of a diotic noise. A main finding was that interaural differences in time and level were independently impaired by various types of loss and, as with localization, the most severe binaural impairment was observed in those with extreme unilateral losses. Furthermore, those people with SNHL and poor speech understanding (who were impaired on the vertical MAA and the side-referenced horizontal MAA) had normal JNDs for time and intensity, as did the participants with similarly impaired audiograms and good speech understanding.

**FIGURE 6:**
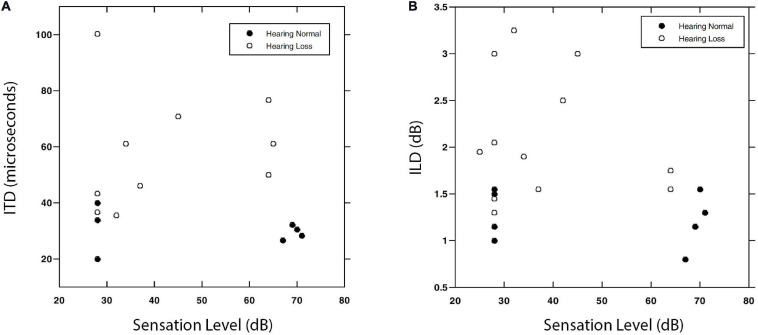
Interaural time difference **(A)** and ILD **(B)** values associated with JND thresholds for a 0.5 kHz narrowband noise. Data are shown for normally hearing (filled symbols) and hearing-impaired (open symbols) listeners as a function of SL. Note that the normally hearing listeners repeated the task at both a high and a low SL, while the impaired group were tested at a single level from which SL was calculated. Reproduced with permission from Smith-Olinde et al. (2004). Copyright 2004, American Speech-Language-Hearing Association.

Hawkins and Wightman (1980); Koehnke et al. (1986), and Smoski and Trahiotis (1986) also used forced-choice methods to test sensitivity to a set of different binaural cues and/or different stimuli for small groups of people with hearing loss. In each case, despite careful training, large numbers of trials, and careful control of stimulus and response variables, there was a trend toward worse performance in the group with hearing loss, but invariably there were some who still performed in or near the normal range. Gabriel et al. (1992); Koehnke et al. (1995), and Smith-Olinde et al. (2004) measured sensitivity to ITD and ILD with a variety of carriers and reference conditions and found substantial binaural impairment in their participants with reduced sensitivity to pure tones, but were unable to find specific relationships between interaural sensitivity and values of the audiogram. Figure 6 reproduces data from Smith-Olinde et al. (2004) for ITD and ILD sensitivity as a function of SL. Neither the normal-hearing nor the hearing-impaired listeners appear to have JNDs that are predictable from SL alone.

While asking listeners to report the location of sounds presented over headphones is a direct method of measuring binaural sensitivity, the MLD has the advantage of simplifying the task to one of detection of a signal in noise, which does not require introspection about binaural percepts and thus may lead to more reliable performance. Melnick and Bilger (1965) conducted one of the first studies of sensitivity to interaural differences in people with peripheral hearing loss and did so by measuring the MLD for a speech target. Inspired by the work of Licklider (1948), they conducted a detailed investigation of the MLD with 61 patients and 14 normally hearing listeners. Despite the presence of hearing losses that ranged from mild to severe and symmetrical to asymmetrical, all of those tested were able to make use of the IPD to obtain better speech intelligibility. Unfortunately for later researchers, the data were only compared in terms of group means (which showed no differences) and rather than reporting the MLD for each listener, patients were simply categorized as “normal” or “abnormal” with reference to normal performance.

Bocca and Antonelli (1976) performed a similar study, but rather than categorical reports of performance, they compared MLDs across groups of patients and control listeners. The MLD was determined for speech signals presented in white noise. Interaural conditions were tested in which the speech and noise were both diotic (N_0_S_0_), or the noise was diotic and speech was delayed to the left or right ear by 0.8 ms (N_0_S_*T*_). The MLD was defined as the difference between the levels that produced the same percentage of correct responses for N_0_S_0_ and N_0_S_*T*_, based on the psychometric functions obtained. Data were compared to that of a control group of 20 listeners with normal hearing thresholds, who had an average MLD of roughly 7.5 dB.

For ten listeners with symmetrical CHL (PTA of at least 60 dB HL in both ears), the MLD was only slightly lower than that of the controls (7 dB). Ten listeners with asymmetrical conductive loss (PTA of 60 dB HL in one ear, PTA of 0–20 dB HL in the other) had an average MLD of 4.5 dB when the poorer ear was leading in time, which was further reduced to 2.5 dB when the better ear was leading. For those with Ménière’s disease (essentially flat unilateral losses of at least 50 dB HL), the MLD was roughly – 0.5 dB when the signal to the poorer ear was leading in time and was 3.5 dB when the better ear was leading in time. For those with presbycusis, the MLD was 6 dB. These results support the findings of Melnick and Bilger (1965) in that MLDs were obtained for all the patient groups in at least one condition, showing that all had the ability to benefit from interaural differences. The differences in MLD based on the ear leading in time suggests that there may be important interactions between the damage and the stimuli, but, as the authors note, the experiments conducted are insufficient to provide insight into all of the issues that were uncovered. It was also unclear why the asymmetrical conductive loss patients had higher MLDs when the poorer ear was leading in time, but the effect was reversed for those with Ménière’s disease. In general, there has been very little work in the past 20 years either on the effects of asymmetrical losses on sensitivity to ITD or on the effects of Ménière’s disease on binaural hearing.

Olsen et al. (1976) also tested the MLD in a range of patients, and were seemingly unaware of the work of Melnick and Bilger (1965). Their study was immediately replicated (Olsen and Noffsinger, 1976) using the same methods. Three conditions were tested: N_0_S_0_, where both signals were diotic, and N_0_S_π_ and N_π_S_0_ and where one signal was diotic and the other was reversed in phase at the two ears. As the methods from the two studies were essentially identical, the results have been combined here. Additional data from this study will be described in the section titled “Studies on Binaural Function With Participants With Central Dysfunction” as well. Combining the patients with peripheral loss across both studies results in a group of 124 patients: 62 with high-frequency noise-induced hearing loss, a group of 32 people with unilateral losses due to Ménière’s disease, 10 patients with unilateral conductive loss, and 20 patients with presbycusis. Results were compared to data from 62 control participants with normal hearing. Ninety-one percent of those with Ménière’s disease showed abnormal performance, as opposed to only 50% of those patients with presbycusis or conductive losses. Thirty-seven percent of those with noise trauma performed abnormally. These results support the conclusion that low-frequency hearing loss, which is more common in Ménière’s disease than in the other groups, is one of the aspects of peripheral hearing loss most likely to result in binaural dysfunction.

**FIGURE 7:**
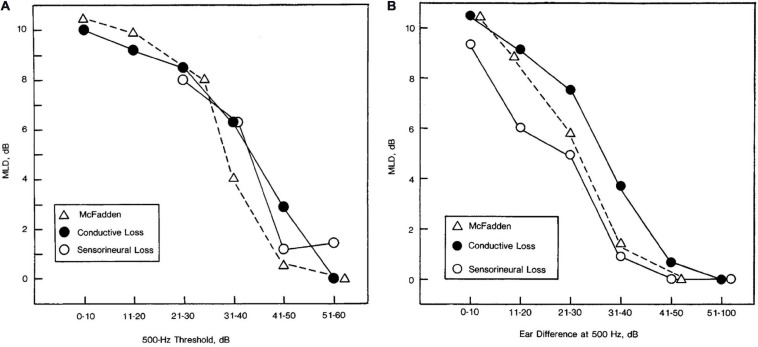
Masking level difference as a function of pure-tone detection threshold at 500 Hz for listeners with symmetrical hearing losses across ears **(A)** and the amount of asymmetry in pure-tone detection thresholds between ears at 500 Hz for listeners with unilateral losses **(B)**. Data are shown for 28 participants with conductive loss in panel **(A)**, 48 participants with conductive loss in panel **(B)**, 71 participants with sensorineural hearing loss in panel **(A)**, and 55 participants with sensorineural hearing loss in panel **(B)**. For comparison purposes, the relevant data from McFadden (1968), in which normally hearing listeners detected signals of various levels are also plotted in each panel. Reproduced with permission from Jerger et al. (1984). Copyright 1984, American Medical Association. All rights reserved.

Jerger et al. (1984) measured the MLD for pure tones in a very large group of people with a wide range of symmetrical and asymmetrical conductive and SNHLs (*n* = 651). The main result, consistent with the data just reviewed, was that the MLD was found to depend on hearing thresholds at 500 Hz and to follow the same audibility function measured by McFadden (1968). Data from that study are reproduced in Figure 7. Jerger et al. (1984) suggested that these curves could be used to adjust the expected MLD values as a function of 500-Hz detection threshold. Adjustments for higher frequency losses and asymmetrical losses were also described. Jerger et al. (1984) suggested that these corrections could be used to identify patients with abnormally small MLDs, and thus the MLD be used to screen for retrocochlear pathologies, as is discussed in the section titled “Studies on Binaural Function With Participants With Central Dysfunction.”

One of the essential insights into both peripheral loss and binaural impairment that has emerged in the past two decades is the idea that peripheral loss can lead not only to reduced sensitivity to pure-tones, but can also interfere with phase-locking at the level of the auditory nerve. Recognition of the importance of sensitivity to both the TFS and the TES of the binaural stimulus has led to significant advancements in both the testing and modeling of binaural impairment. For example, Lacher-Fougère and Demany (2005) showed that hearing impairment reduces sensitivity to ITD in the carrier but not the envelope of modulated tones, implying impaired TFS processing but preserved TES processing, and only a weak relationship with the audiogram. Similarly, the influence of hearing loss on the MLD (as shown in Figure 7) has recently been replicated and expanded with extensive modeling by Bernstein and Trahiotis (2016, 2018, 2019), who demonstrated that even very small changes in pure-tone detection threshold can result in reliable reductions in the MLD that can be modeled by reduced encoding of TFS. Moore (2020) provides a comprehensive overview of these results as well as a range of other recent studies of the role of TFS in binaural sensitivity (e.g., Ross et al., 2007; Hopkins and Moore, 2009; Grose and Mamo, 2010; King et al., 2014; Füllgrabe and Moore, 2018).

### Peripheral Loss and SRM

Another area in which the effects of peripheral loss have been studied extensively but are still poorly understood mechanistically is that of SRM, which is defined as any improvement in target detection or recognition that accompanies the introduction of spatial cues that differ between a stimulus to be detected or identified (the target) and a stimulus to be ignored (the masker). Binaural release from masking (BRM) refers to the improvements that occur when a binaural cue is provided, which may or may not result in a spatial percept. It is worth distinguishing the two, especially when the goal is to understand the underlying mechanisms.

Early work, described in the section on peripheral loss and binaural sensitivity to interaural differences, focused on the MLD, using headphone presentation in the presence of noise (Olsen et al., 1976; Olsen and Noffsinger, 1976). Much of the later work (Duquesnoy, 1983; Bronkhorst and Plomp, 1989; Peissig and Kollmeier, 1997; Arbogast et al., 2005) moved to loudspeaker presentation of target and masker in various spatial configurations, as well as introducing the use of speech as a masker. While these modifications increased the realism of the testing scenarios, in these studies the maskers were generally presented from a single location, resulting in different SNRs at the two ears. This in turn leads to the availability of a “better-ear” listening strategy, which means that performance may improve even if the listener has no binaural sensitivity whatsoever. Using a method suggested by the manipulations and better-ear calculations of Hawley et al. (2004); Marrone et al. (2008) demonstrated that the better-ear effect can be significantly reduced (at least in terms of the long-term spectrum) by displacing two maskers symmetrically to the left and right of the target. In these conditions, people with higher pure-tone detection thresholds still exhibit less SRM than do people with thresholds in the normal range. Gallun et al. (2013) introduced a rapid version of this test and observed independent effects of age and hearing loss on performance, but testing was limited to those with SNHL in the mild to moderate range. The test can also be performed using a virtual loudspeaker array presented over headphones with similar results (Jakien et al., 2017; Srinivasan et al., 2020), which allowed Ellinger et al. (2017) to use the VAS to present processed speech signals that included ITD, ILD, or both. Ellinger et al. (2017) found that people with higher pure-tone detection thresholds due to SNHL obtained spatial benefit in all of the conditions tested. Jakien and Gallun (2018) used symmetrical maskers to test a large sample of listeners (*n* = 82) varying in age, with and without mild-to-moderate SNHL. The results were used to make a predictive linear regression model of performance which was able to account for 38% of the variance in SRM with only the audiogram. Kubiak et al. (2020) used a more sophisticated model, incorporating speech intelligibility measured with standard diagnostic tests, and were able to account for up to 80% of the variance in speech intelligibility among a group of 23 participants with impaired hearing due to SNHL and 7 with normal hearing. These results suggest that while there is significant variance unaccounted for by the pure-tone detection thresholds, perhaps some of the equivocal results of earlier work were due to insufficiently large sample sizes or insufficient model complexity.

Other studies with symmetrically placed maskers (Glyde et al., 2013; Besser et al., 2015) have also found a relationship with pure-tone detection thresholds. Best et al. (2013, 2017) have argued, however, that this relationship may be due to an “energetic limit” on spatial release, where people with more impaired hearing require higher SNRs to understand the target. Based on this argument, the relationship with pure-tone detection threshold may be epiphenomenal, and due to the reduced performance at low SNRs, rather than a binaural deficit. This again argues for the importance of relying less on indirect measures of impairment, such as pure-tone detection thresholds, and relating performance in real-world environments to specific tests of binaural sensitivity.

**FIGURE 8:**
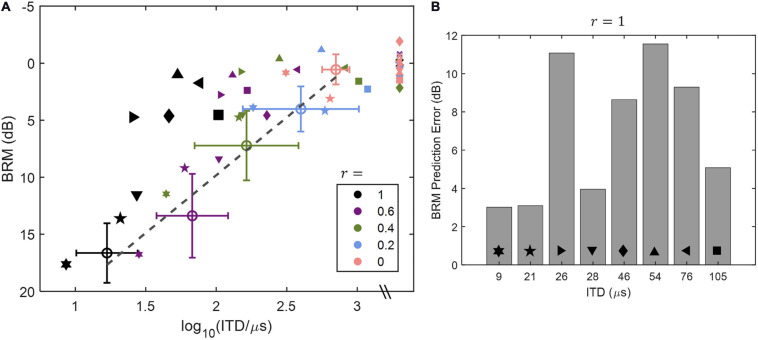
Data and modeling reproduced with permission from Baltzell et al. (2020). Copyright 2020, Acoustical Society of America. Relationships between ITD threshold and BRM as a function of interaural correlation (*r*) of the stimuli tested are shown in panel **(A)**, and the deviation in dB between the best-fitting line for the listeners with normal hearing [open circles in panel **(A)**] and the values for the hearing-impaired listeners [filled symbols in panel **(A)**] are plotted in panel **(B)**. Values of *r* are indicated by color of the symbols in panel **(A)**, and gray symbols indicate points for which the ITD was unmeasurable. One listener, who had an unmeasurable threshold for an interaural correlation of 1, was excluded from the analysis and is not included in the figure. See text for further details.

One study that has applied such a direct approach is Baltzell et al. (2020), who used a headphone test to measure ITD sensitivity and BRM with only an ITD cue for 11 normally hearing listeners and 9 listeners with impaired hearing. By manipulating the interaural correlation of the stimuli for both tests, they were able to obtain a range of ITD thresholds and a range of BRM values for all participants. The relationships between ITD threshold and BRM that were observed are plotted in Figure 8A. While BRM was well-predicted by ITD sensitivity in listeners with normal hearing, there was great variability among those with impaired pure-tone thresholds, and many had BRM values that were worse than were predicted by the best-fitting line for the control participants. Figure 8B shows the deviation from the predictions for the listeners with impaired hearing. In addition, as has been found by others (Jerger et al., 1984; Neher et al., 2011, 2017; Bernstein and Trahiotis, 2016, 2018, 2019; King et al., 2017), low-frequency PTA was a significant predictor both of ITD sensitivity and of BRM.

From the data presented in sections “Peripheral Loss and Binaural Sensitivity to Interaural Differences” and “Peripheral Loss and SRM,” it is clear that at least some listeners with poorer pure-tone detection thresholds are impaired on tasks requiring good TFS sensitivity, especially for narrowband stimuli and those in which the ITD is not present in the TES (Grose and Mamo, 2012; Gallun et al., 2014; Spencer et al., 2016; Best and Swaminathan, 2019; Baltzell et al., 2020). On the other hand, there are many participants described in the literature with binaural sensitivity in the normal range, despite poor ability to detect pure tones at low levels. As some of the early literature suffers from methodological issues, it might be tempting to argue that the differences are based on methodology of the experiments or perhaps motivation and/or training of the listeners. In order to address this Spencer et al. (2016) went to great lengths to collect a data set using the strongest methods, including training the listeners extensively and repeating the measures to ensure that only data with strong internal consistency were included. Indeed, internal consistency was high across the full data set, suggesting that the values were measured reliably. Nonetheless, substantial heterogeneity was observed among the younger hearing-impaired listeners tested, with many performing in the same range as the normal-hearing control participants. The results of Spencer et al. (2016) underscore the message of nearly 100 years of research on hearing impairment and sensitivity to interaural differences: the ability to detect pure-tones is an indicator of who may be suffering from binaural impairment, but it is not sufficient for strong predictions, especially when the stimuli are broadband and the hearing loss is worst in the high frequencies.

The modeling approaches that have been most successful in predicting the binaural abilities of individual listeners (rather than group differences) have combined pure-tone detection thresholds with metrics unrelated to spatial cues such as age, measures of speech understanding (Kubiak et al., 2020), and/or measures of cognitive function (Gallun and Jakien, 2019). In addition, there are computational modeling approaches that show great promise in helping identify the specific mechanisms responsible for binaural impairment (Le Goff et al., 2013; Mao et al., 2015; Moncada-Torres et al., 2018). The most promising opportunities for future research are those that involve a process of informational feedback between human patient research and targeted animal and computational models. Ideally, such a program would start by developing models based on the existing human data, which in turn would predict the factors that are most important for measuring in the patients. Then the human experiments could be developed to measure and control those factors as a way of testing the models. To date, few binaural clinical research programs have followed this process, but some of the most successful (e.g., Baltzell et al., 2020) are definitely moving in this direction.

## Studies on Binaural Function With Participants With Central Dysfunction

While many researchers have focused their studies of binaural impairment on the relationship with pure-tone detection thresholds, as audiological practice might suggest, there are a number of other groups that have conducted investigations of some of the other ways in which binaural function could be impaired. Much of the earliest work on binaural impairment was conducted with patients with normal or near-normal pure-tone detection thresholds who were diagnosed with MS, strokes, brain tumors, or traumatic brain injury. More recently, there has been considerable interest in the effects of aging, alone and in combination with hearing loss, on binaural function. The results of such studies are extremely important for connecting the animal literature on binaural processing to the human literature on binaural function. In animals, detailed information can be gained about the pathways and signal processing associated with the binaural system, but data on complex behavioral tasks are very difficult to obtain. With humans, the opposite is generally true. The exception is when imaging data are available showing precise lesion locations for a patient who has also performed spatial hearing tasks. As imaging techniques improve and our understanding of neurological disease progresses, there is great opportunity for our knowledge of the mechanisms of binaural hearing to improve as well.

### Aging

One of the most difficult issues associated with the study of hearing loss, especially in the high frequencies, is the comorbidity with aging. For binaural impairment, this issue is especially important to address, as there is considerable reason to believe that those with aging auditory systems can potentially suffer from a wide range of monaural and cognitive impairments that are likely to influence performance on tests of binaural function (reviewed in Gallun and Best, 2020). In addition, it is increasingly clear that aging itself can impair the functioning of the binaural system. One of the major obstacles to studying the effects of aging on binaural processing is the difficulty of comparing younger and older listeners independently of differences in peripheral hearing. Either the researcher must limit participants to a very specific audiogram, which may limit the generalizability of the results, or statistical approaches must be used to factor out the effects of peripheral hearing loss. Statistical separation of the influences of the two factors requires testing larger samples and recruiting listeners in a manner that age and hearing loss can be statistically dissociated.

#### Aging and Localization

**FIGURE 9:**
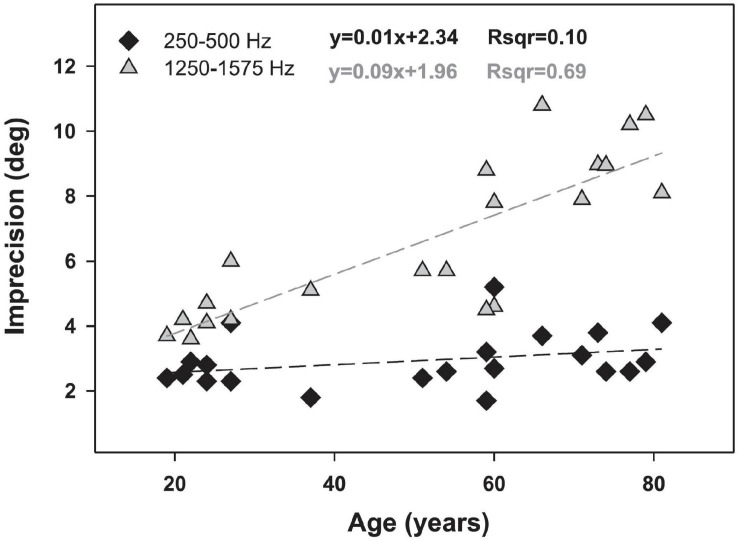
The relationship between age and localization accuracy for two narrowband signals: 250–500 Hz (black diamonds) and 1,250–1,575 Hz (gray triangles). Dotted lines indicate linear regressions associated with the equations shown. Data and modeling reproduced with permission from Dobreva et al. (2011). Copyright 2011, American Physiological Society.

Abel et al. (2000) tested the horizontal localization abilities of 112 participants aged 10–81, divided into seven age groups, each with 16 participants within the same decade of life. Both of the youngest groups had hearing thresholds no greater than 12 dB HL from 0.5 to 4 kHz, but those aged 30–59 were allowed to have thresholds up to 22 dB HL and those 60–81 were allowed to have thresholds has high as 37 dB HL. Thus, there was a systematic increase in average thresholds with age, reaching a maximum difference of 25 dB at 4 kHz between the youngest and oldest groups. Horizontal localization performance declined significantly by the third decade and overall was reduced by 12–15% between the youngest and the oldest listeners. Much of the error was attributable to front-back confusions, however. As these errors are likely to be associated with loss of spectral cues due to high-frequency audibility differences between the groups, it is not possible to say definitively whether these data represent an aging effect independent of the age-related changes in hearing thresholds. Similar studies by Dobreva et al. (2011) and those reviewed by Freigang et al. (2015) also found that the accuracy and precision of localization is reduced in older listeners, but it was not possible to definitively separate the effects of aging from slight age-related declines in peripheral function. The effect of age on localization is shown in Figure 9, reproduced from Dobreva et al. (2011). While these results show a strong effect of aging on horizontal localization for mid-frequency signals, but not low-frequency signals, they are also consistent with the results of Bernstein and Trahiotis (2016, 2018, 2019), who suggested that horizontal localization ability may be reduced by hearing loss within the normal range. In this case it is difficult to determine whether these changes in localization ability are truly age effects or whether they might be the effects of small differences in detection ability.

#### Aging and Binaural Sensitivity

**FIGURE 10:**
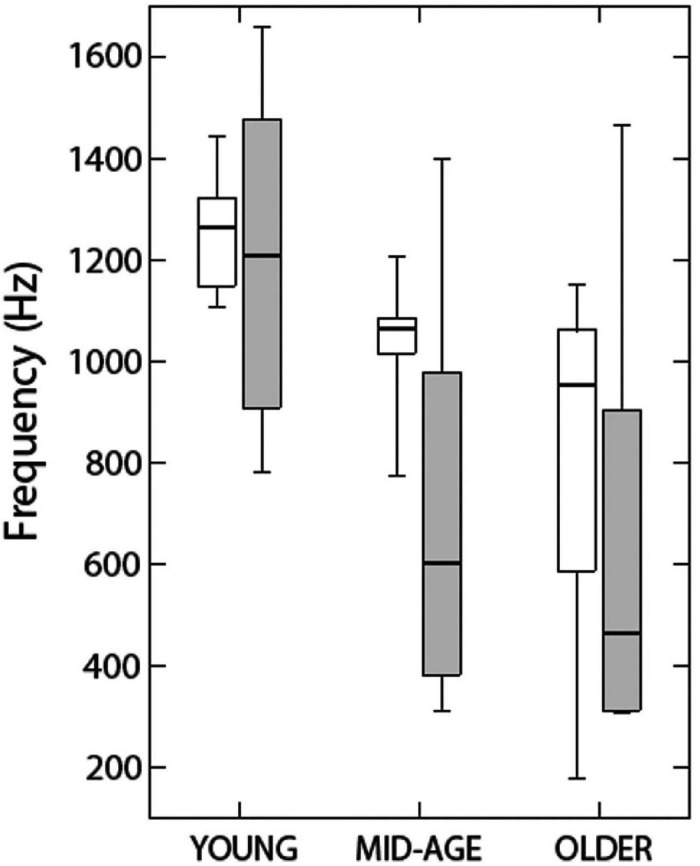
Data indicate the highest carrier frequency at which listeners in three age groups could discriminate diotic from dichotic stimuli. Open rectangles show data from Grose and Mamo (2010) and shaded rectangles show data from Ross et al. (2007). See text for further details. Reproduced with permission from Grose and Mamo (2010). Copyright 2010, Wolters Kluwer Health, Inc.

Ross et al. (2007) measured the effects of aging on binaural function using both behavioral and magnetoencephalographic measures. They controlled for hearing effects by ensuring that all listeners had thresholds below 20 dB HL between 0.5 and 2 kHz and no higher than 40 dB HL at 4 kHz. As mentioned in the section on aging and localization, however, this does not allow us to rule out the influence of small differences in audibility. Their behavioral measure showed that the middle-aged and older listeners were less able to detect an interaural phase shift in an ongoing amplitude-modulated tone than were the younger participants in the study and, as will be discussed in the section on neural measures of binaural sensitivity in older listeners, that this difference was reflected in recording of their brain activity. Their behavioral results were replicated and extended by Grose and Mamo (2010, 2012). The results of Grose and Mamo (2010; Figure 10) showed that while 60% of the listeners younger than 27 years were able to detect phase shifts for stimuli with carrier frequencies as high as 1.25 kHz, only 15% those 40–55 years of age could do the task at this frequency, and only about 5% of the listeners who were aged 63–75. Grose and Mamo (2012) extended these results by using the dichotic FM task described in the section on methods of characterizing binaural impairment, in which a 500-Hz tone was modulated in frequency in different directions in the two ears, creating a fluctuating IPD. The same range of hearing losses were present in these listeners. While the younger group (aged 19–29 years, *n* = 12) could detect binaural FM of 0.4 Hz on average, the middle-aged group (43–57 years) could only detect binaural FM of 0.8 Hz, and the older group (aged 65–77 years) needed almost 2 Hz of modulation before they could perform the task. The younger group also performed better than the other two groups when the stimuli were presented diotically, but even the younger listeners still needed more than 2 Hz of modulation in order to perform the task with no binaural difference. When the stimuli were presented diotically to the middle-aged and older groups, they needed 3 and 3.5 Hz, respectively.

King et al. (2014) tested 46 listeners varying in age from 18 to 83 with a wide range of pure-tone detection thresholds (−1 to 68 dB SPL at 1 kHz) for whom age and pure-tone sensitivity was uncorrelated at 0.5 and 1 kHz (*r* = 0.08) but more strongly correlated at higher frequencies (*r* = 0.439). Listeners were asked to detect IPDs in low frequency (250 or 500 Hz) tones amplitude-modulated at a rate of 20 Hz. IPDs were applied to either the carrier or the modulator in order to test the hypothesis that there is an age-related deficit in TFS processing that is independent of a binaural impairment. Presentation levels were set by first measuring the detection threshold for the stimuli and presenting all stimuli at a minimum of 30 dB SL. The data revealed age-related deficits in binaural processing for the 500 Hz tones, whether the IPD was applied to the carrier or the modulator, and for the 250 Hz tones, but only when the IPD was applied to the modulator. These data were taken to support a generalized age-related decline in temporal processing rather than a specifically binaural impairment.

Füllgrabe (2013) also found evidence for a general temporal processing deficit in a sample of 102 participants with normal audiometric thresholds varying in age from 18 to 90 years. Both a monaural TFS test and a binaural TFS test showed systematic declines in performance as age increased. Füllgrabe and Moore (2018) performed a meta-analysis of 19 studies that used the same binaural TFS test and found that while age and pure-tone detection thresholds were both significant predictors of performance, age accounted for more variance in every comparison conducted. The total amount of variance accounted for by both factors was never more than 42%, however, suggesting that these two variables alone are insufficient to account for performance on even a very specific psychophysical task.

Whiteford et al. (2017) also measured the effects of age on monaural and binaural temporal sensitivity by comparing the detection of slow-rate (1 Hz) AM and FM with the detection of fast-rate (20 Hz) AM and FM. Both AM and FM were tested diotically and dichotically. Dichotic AM results in time-varying ILDs and dichotic FM results in time-varying ITDs, as described above for the experiments of Grose and Mamo (2012). Whiteford et al. (2017) tested 85 listeners aged 20–80 years, with pure-tone average thresholds (0.5, 1, and 2 kHz) that were all in the normal range (no greater than 20 dB HL). Average thresholds and age were correlated (*r* = 0.56). All stimuli were presented at 60 dB SPL. The hypothesis tested was that there would be at most small effects of age on AM detection at either rate, but that FM detection would be more impaired with aging for the slow-rate stimulus, where only TFS cues were available. Contrary to expectations, however, age effects were observed not only for both slow and fast FM presented diotically or dichotically, but also for fast dichotic AM. These results also support the idea that impairments in temporal processing associated with aging are likely to involve a variety of processes, including, but not limited to, TFS sensitivity.

**FIGURE 11:**
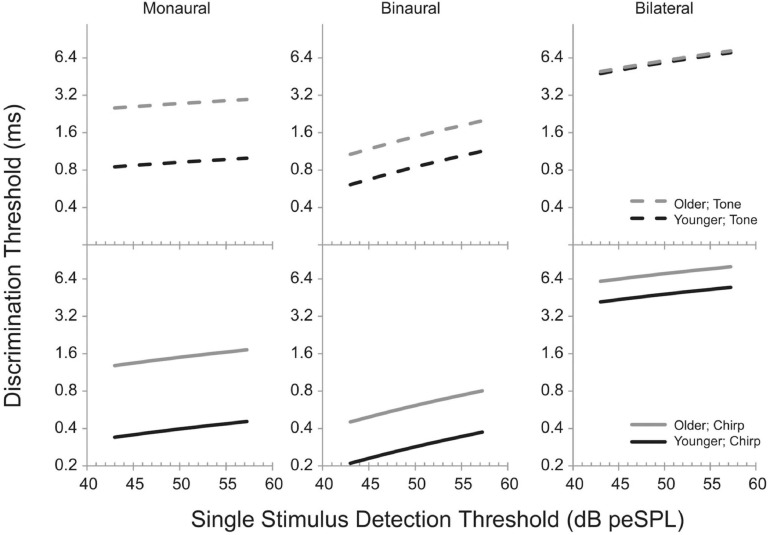
Model predictions from Gallun et al. (2014) showing the effects of detection threshold for a brief (4 ms) stimulus in peak-equivalent units (peSPL; equivalent level for a 1-s pure tone with the same peak level) on discrimination thresholds. Predictions are shown for a modeled younger listener (20 years; black lines) and a modeled older listener (gray lines). Solid lines are for a broadband stimulus (“chirp”) and dashed lines are for a narrowband stimulus (“tone”). See text for a description of the monaural, binaural, and bilateral tasks. Reproduced with permission from Gallun et al. (2014) under Creative Commons license.

Gallun et al. (2014) reached similar conclusions when they measured the temporal processing abilities of a large group of participants (*n* = 78) varying in age (18–75 years) and hearing loss (0–40 dB HL at 2 kHz). Listeners were tested on monaural, binaural, and bilateral timing discrimination tasks with brief (4 ms) stimuli with narrow or broad frequency content, all centered at 2 kHz. In the monaural task, listeners were asked to detect a gap between two stimuli, in the binaural task they were asked to detect an ITD, and in the bilateral task, they detected delays between the presentation of a signal to the left and right ear. Gaps, ITDs, and bilateral delays were adaptively varied to determine threshold. Presentation level was set to 30 dB above detection threshold for each stimulus. Rather than relying upon group differences or partial correlations, a linear mixed model was developed in which performance across all stimuli and tasks was modeled, taking into account each individual’s age and stimulus detection thresholds. One advantage of this approach is that each individual can be assigned an intercept value for their function, reflecting that individual’s ability to perform psychophysical tasks. The model predicted 20–40% increases in monaural gap detection thresholds per decade of aging, 15–20% increases in ITD discrimination thresholds with every decade, and 0.9–10% increases in bilateral delay sensitivity, all independently of increases in temporal processing ability with increases in signal detection thresholds. Figure 11 shows the model predictions from Gallun et al. (2014), where the age effects are indicated by the predictions for a 20 year old (black lines) and a 60 year old (gray lines). The top row shows the increases in threshold in the three conditions for a tonal stimulus as a function of increases in stimulus detection thresholds, while the bottom row shows the changes predicted for the same listeners and conditions for a broadband stimulus. Figure 11 demonstrates that while there are indeed timing and/or binaural impairments associated with even slight hearing loss, statistical modeling can be used to more clearly quantify the independent effects of aging and pure-tone sensitivity and their interactions with stimuli and tasks.

#### Aging and SRM

Many of the studies examining the effects of hearing loss on SRM, especially with speech stimuli, have also focused on the effects of aging. Early work measuring the MLD with tones (e.g., Olsen et al., 1976) was replicated by Pichora-Fuller and Schneider (1991, 1992), who found that older listeners with slight hearing loss had significantly impaired MLDs and were able to account for this by applying a computational model in which TFS sensitivity varied for younger and older listeners. Duquesnoy (1983) and Gelfand et al. (1988) both noted reduced spatial benefit in their older participants with normal hearing relative to their younger participants. However, the effects were smaller than were the differences between the participants with normal and impaired hearing.

Dubno et al. (2008) were the first to use symmetrically placed maskers and compare younger and older listeners with normal hearing (*n* = 30). While SRM did not differ between the groups, performance for the older listeners was worse than a model based on the audiogram predicted, and there was a strong correlation between age and performance in the spatially separated condition. Marrone et al. (2008) also tested younger and older listeners with and without hearing impairment (*n* = 40) and observed relatively small effects of age independent of hearing loss. Glyde et al. (2013) measured SRM in a speech-on-speech masking task with a group of 80 listeners varying in age (7–89 years) and hearing loss. While there were substantial effects of age in the simple correlations, partial correlations taking into account hearing loss did not reveal significant relationships between age and performance. One possible reason for this is the inclusion of children, for whom spatial benefit increases with age, rather than declining as it does for adults.

Gallun et al. (2013) used a version of the Marrone et al. (2008) task and tested 52 listeners across three experiments and showed stronger effects of aging than of hearing loss on SRM. One possible reason for the difference between these results and those of Glyde et al. (2013) was the use of 45° of separation between target and each masker as opposed to the 90° used in Glyde et al. (2013). Jakien et al. (2017) verified that the SRM observed in the Marrone et al. (2008) task is maximal at about 45°, suggesting that even an impairment that reduces the “effective” spatial separation from 90° to 45° would likely have a minimal effect on SRM. Srinivasan et al. (2016) explored the effect of spatial separation in greater detail by examining SRM with small separations and discovered that the effects of aging are the most apparent with separations less than 15°.

These results with SRM support the findings of age-related declines in binaural sensitivity and localization accuracy. The primary difficulty with interpreting all of these results, however, is that it is unclear whether the mechanisms by which aging and hearing loss cause binaural impairment are fundamentally different. Future work combining human studies with computational models of the binaural system can shed light on this, especially if informed by animal models of binaural impairments associated with age and hearing loss.

#### Neural Measures of Binaural Sensitivity in Older Listeners

**FIGURE 12:**
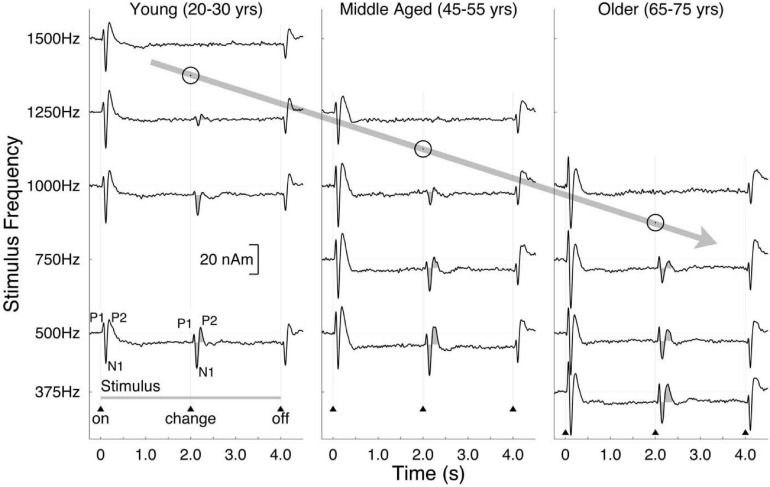
Auditory evoked responses measured by MEG showing the P1-N1-P2 complex to a change from a diotic to a dichotic stimulus as a function of carrier frequency and age group. Data reproduced with permission from Ross et al. (2007). Copyright 2007, Society for Neuroscience.

As mentioned in the section on aging and binaural sensitivity, Ross et al. (2007) conducted the first study to compare older and younger listeners on both behavioral and neural measures of binaural sensitivity. Using the P1-N1-P2 complex responses as measured with MEG (see section “Methods of Characterizing Binaural Impairment” for details) as well as a behavioral detection task using the same stimulus, they were able to compare the highest carrier frequency at which an interaural phase reversal in the carrier frequency of an amplitude-modulated tone was detectable by a human observer and at which the P1-N1-P2 complex was detectable. Both the maximum frequency at which the P1-N1-P2 complex was present and the maximum frequency at which the older listener could detect the binaural change was lower for the older listeners than for the younger, and even the middle-aged listeners differed from the younger listeners. Data from Ross et al. (2007) are shown in Figure 12. These results led to a substantial increase in the number of researchers interested in the effects of aging on binaural sensitivity. Many of the studies that followed (e.g., Papesh et al., 2017; Eddins and Eddins, 2018; Eddins et al., 2018; Vercammen et al., 2018) suggested that age-related binaural impairment on behavioral tasks in humans is related to reductions in TFS encoding. Eddins and Eddins (2018), based on the finding that binaural encoding is reduced for a 500 Hz stimulus but not a 4,000 Hz stimulus, suggested that TFS encoding at the level of the cortex is reduced by aging but that TES sensitivity is not. While the electrophysiological and behavioral responses at the level of the cortex are fairly strong for these and other binaural tasks (Papesh et al., 2017), some electrophysiological measures (Anderson et al., 2018) show poor relationships with binaural sensitivity. In addition, some research (Koerner et al., 2020) has found a diversity of relationships between neurophysiological responses and different behavioral tasks in the same listeners. Koerner et al. (2020) were the first to use a behavioral task that used a stimulus that was directly comparable to the IPM-FR stimulus and found no relationship between the behavioral results and aging or behavioral thresholds and neural amplitudes. In the same listeners, however, the SRM task used by Papesh et al. (2017) was correlated with the amplitude of the neural response at an individual level.

Anderson et al. (2018) interpreted the lack of a relationship between the behavioral and neural data in their study as suggesting that the relationship between encoding and behavior may be more complicated than simply that aging leads to increased variability in TFS encoding at the level of the auditory nerve, which should lead to decreased behavioral thresholds that are related to reduced neural amplitudes. Consistent with this interpretation, electrophysiological recordings in aged monkeys (Juarez-Salinas et al., 2010; Engle and Recanzone, 2013) revealed age-related degradations of the inhibitory connections between cortical and subcortical brain areas, leading to reductions in the tuning of cortical areas specialized for spatial hearing. If this occurs in the brains of older humans as well, then the brainstem encoding could be reduced by one mechanism (perhaps related to auditory nerve phase locking) while the cortical representations of space could be reduced by a separate mechanism (perhaps related to reduced inhibitory connections between brain areas). On the other hand, Maier et al. (2008) tested older gerbils on a spatial discrimination task and found reduced performance, but in this case accompanied by reduced inhibition within the brainstem structures essential for processing interaural timing differences.

In summary, our understanding of the mechanisms relating aging to binaural sensitivity is far from complete. What is clear is that there are aging effects that can be observed both behaviorally and neurally, and that animal and computational models have the potential to both clarify existing data and point the way toward new approaches to the study of aging and binaural function.

### Multiple Sclerosis and Binaural Impairment

Multiple sclerosis is a disease of the nervous system that leads to lesions both at the level of the brainstem and at the cortex. While there have been a small number of studies of binaural sensitivity in MS patients, the promise of modern imaging technologies has only begun to be explored. With the ability to identify specific lesions and relate them to binaural function, it may be possible to obtain evidence in humans of the specific roles of various brain areas; evidence that only animal models could previously provide. This is also the promise of the work detailed in the section on the effects of brain tumors and lesions on binaural sensitivity.

The existing literature shows clearly that MS patients are likely to have binaural deficits. One of the earliest reports to show this was the MLD data on 100 patients with MS reported by Olsen et al. (1976), “almost all of whom” had normal audiograms and speech reception thresholds in quiet. Using both speech and tonal targets and comparing to the MLD from a group of control subjects, at least 41% percent of the MS patients were in the abnormal region for one or more stimuli. Jerger et al. (1986), using the correction to the MLD for hearing thresholds developed in Jerger et al. (1984), found the MLD to be abnormal in 45% of a group of 62 MS patients. Similarly, Musiek et al. (1989) found that 50% of a group of 26 MS patients (all with normal audiograms) had abnormal MLDs, as compared with 20 control subjects.

In addition to the MLD, some studies have also examined sensitivity to ITD and ILD directly, such as Häusler and Levine (1980), who measured JNDs in interaural time and intensity for 29 patients with MS. Results were compared to those of 36 normal-hearing controls with no known neurological impairment. All of the controls had ITD JNDs between 10 and 40 μs, while 13 of the MS patients had ITD JNDs of 50 μs or greater. All of the controls had ILD JNDs of 0.5 – 2.0 dB, while 6 of the MS patients had thresholds of 2 dB or greater. Similarly, Häusler et al. (1983) included 26 of the patients with MS in their study. Of these, two-thirds had abnormal performance with the side referenced MAA task but did well on the center-referenced MAA task. Colburn (1982), in reviewing these data stated:

“The fact that different tests (particularly interaural time and interaural intensity discrimination) give independently normal and abnormal performance, even with the same stimulus, suggests that different regions of the brain are involved in processing the different aspects of the stimulus, such as interaural time delay versus interaural intensity differences” (p. 42).

This statement reveals how useful it can be to test patients with neurological disorders and how essential it is to pair these tests with animal models and the results of neurophysiological experiments. While it is well-accepted today that ITD and ILD are processed by independent brain regions, these patient data were early indicators that preceded the more definitive neurophysiological studies to come.

In the 1990s, several additional studies of lateralization and sensitivity to interaural differences were conducted with patients suffering from MS (Levine et al., 1993a, b, 1994; Furst et al., 1995; Häusler and Levine, 2000). Due to improvements in the ability to image the central auditory system, these studies were able to shed light on how the MS lesions related to binaural impairment. In patients with lesions of the brainstem, binaural function was found to be impaired on both lateralization and detection of interaural differences (Furst et al., 2000). Based on the patterns of MS lesions and their relationships to binaural dysfunction, Furst et al. (2000) proposed a model in which MS impairs ITD-based detection and lateralization due to the imposition of additional conduction delays on the neural networks underpinning ITD sensitivity. Further work in this area would be tremendously informative.

### Binaural Impairment in Patients With Brain Tumors and Lesions

While it is tempting to imagine that the earliest work on binaural impairment focused primarily on peripheral hearing loss, the reality is that Greene (1929) also used his “short-circuited” binaural stethoscope to study fifteen patients with brain tumors. Five of the fifteen were abnormal, as compared to the control group, which distinguished them from those with peripheral loss, none of whom were abnormal in their sensitivity to binaural differences measured in this manner. It was these data that convinced Greene (1929) find collaborators to help him develop the innovative equipment for distinguishing ITD and ILD sensitivity described in the section on methods of characterizing binaural impairment. Using this apparatus, Greene, who was a neurosurgeon, tested 51 of his patients with neurological disease and compared their performance to that of control participants with no known neurological disease and normal peripheral hearing. Only three of the patients showed localization that differed from the control group when they were asked to identify the location of a ticking watch, but fifteen of those with normal localization in the ticking watch task, where both ITD and ILD cues were present, had abnormal lateralization perception with ITD or ILD alone. For ten of these patients, perception of only one cue was abnormal, while for five of them, perception of both cues were abnormal in isolation, despite no impairment when asked to localize a ticking watch.

Greene (1929) used these clinical data to form hypotheses about the underlying physiology, noting that at that time it was unknown whether or not the auditory nerves from the two ears connect to one or both temporal lobes. Based on the observation that the majority of those with impaired localization had temporal lobe tumors, he concluded that it is likely that the monaural auditory pathways extended to both temporal lobes. Furthermore, he mentioned that it was unknown where in the auditory system sound localization occurs. Based again on his finding that the majority of the lesions in those with impairment were in the temporal lobe, he concluded that it is likely that this is where the localization ability resides. He acknowledged, however, that his sample size was too small for this to be more than speculation.

Yet, despite these remarkable aspects, some important elements are missing from Greene (1929). Durlach et al. (1981) pointed out that the data are all reported entirely in terms of average response, with no indication of within-subject variability. In addition, the between-subject information is reported in tables where each listener’s ability to localize is categorized on a six-element scale ranging from “normal” to “completely absent.” It is difficult to read the report and not wish one had access to the full data set upon which these categorical judgments are based.

Walsh (1957) conducted a series of experiments following up on the work of Greene (1929) almost 30 years later, but that took advantage of the advances in electronic devices and in psychophysical techniques that had occurred during the intervening years. The work was inspired by Wallach et al. (1949) and used electrical pulses delivered either to the two ears or to two loudspeakers. A phase-delay circuit was also used to deliver a 250-Hz tone to the two ears either delayed by 90° or undelayed (presented “diotically”). An unspecified “small number” of normal hearing controls were used to establish that the clicks sounded like a single click when the delay was less than 2.5 ms (“precedence threshold”), and that ITDs of 100 μs were about the smallest that could be distinguished from a diotic signal (“ITD threshold”). Twenty-one patients with cerebral lesions were tested, and all could detect ITDs, but roughly half had detection thresholds greater than 300 μs. Several patients were able to distinguish ITDs below 300 μs despite significant lesions to the auditory processing areas of one hemisphere, leading Walsh (1957) to conclude that binaural detection does not require both hemispheres. Of the 15 cerebral lesion patients who completed precedence-effect testing, all reported that for some portion of the range of delay they experienced a single sound (“fusion”) and only four had precedence thresholds exceeding 4 ms. Only one of the 12 cerebral lesion patients who performed the task of detecting a phase-delay was unable to do so successfully. From these studies, Walsh (1957) concluded that it is likely that binaural comparison occurs at the level of the brainstem, the output of which is sent to both hemispheres.

Häusler et al. (1983) did not measure the abilities of people with cerebral tumors, but they did report data from 7 patients who had a tumor on the eighth nerve called a vestibular schwannoma (referred to in the text as a “neurinoma”). Quite uniformly, it was found that these patients were among the most impaired of all the groups tested in that study. All were unable to perform the MAA task in the normal range, in either the center- or side-referenced condition. ITD discrimination thresholds were in the abnormal range for the majority (but not all) of the patients with tumors and only one patient with an eighth-nerve tumor had normal ILD sensitivity. It should be noted that this was the most recent study that could be found in which patients with eighth-nerve tumors were included in a binaural experiment.

Colburn (1982) concluded that for the patients with eighth-nerve tumors,

“information flow on the auditory nerve is extremely disrupted by some auditory nerve lesions. The timing and intensity information can be essentially eliminated at supra-threshold levels, even when the threshold value is only slightly affected. If one postulates a tumor pressing against nerve fibers, it is easy to imagine not only a disruption of the timing of individual action potentials but also an interference with the number of firings (e.g., by an increase in the refractory period) without an associated change in the absolute detection threshold. I am not aware of an animal model for this condition” (p. 41).

It is probably fair to say that little has changed in terms of our understanding of the effects of eighth-nerve tumors on binaural hearing in the intervening decades. On the other hand, one area that has been very informative is the study of patients with strokes that impinge on the auditory brain regions. Furst et al. (2000) reported results from patients with strokes in the auditory brainstem areas and that both lateralization and sensitivity to interaural differences were impaired. From this, they proposed a model in which strokes lead to diminished ITD and ILD, as well as lateralization, by damaging the connections in the brainstem.

Horizontal localization in patients with cortical and brainstem damage from strokes was also studied by Sonoda et al. (2001) and Przewoźny et al. (2015a,2015b), who both found accuracy of sound source identification to be reduced in some of their patients. An important consideration in studying stroke patients is the potential for damage to non-auditory areas to result in difficulties with a localization task. For example, recent work on the relationships between auditory neglect due to stroke and spatial hearing is reviewed in Gutschalk and Dykstra (2015), who conclude that more work is needed to develop clinical protocols that can clearly distinguish localization deficits from disorders of spatial cognition.

### Binaural Impairment in Patients With Traumatic Brain Injury

The work of the Vietnam Head Injury Study (VHIS; Sedge, 1987) stands essentially alone in the study of binaural impairment in those with penetrating head wounds. Phase 2 of the VHIS started in 1980 and tested 482 head injured patients and 82 controls. Most of the tests took place 14 years after injury. Mueller and Beck (1987) reported on the MLD thresholds for a 500 Hz tone presented in narrowband noise obtained from 55 control subjects and 92 Veterans with a history of penetrating head wounds. There was a small difference between the MLD for the controls and those with a history of brain injury, but it was less than 1 dB and was non-significant. These results were interpreted as consistent with the fact that the Veterans in this study had primarily cortical injuries, as the authors believed the MLD to be a measure of brainstem integrity. This is an important finding and one on which it would be useful to have more data.

Another population found to have difficulties with binaural tasks is those who have experienced head trauma (Gallun et al., 2012; Saunders et al., 2015; Roup and Powell, 2016; Hoover et al., 2017; Kubli et al., 2018). Binaural dysfunction in this group is particularly difficult to characterize due to both the heterogeneity of the injuries and the increased likelihood of impairment on complex tasks. The issue of heterogeneity derives in part from the diversity of physical events that can cause even mild Traumatic Brain Injury (mTBI). The literature includes both studies of patients with a history of exposure to high-intensity blasts during their military exposure (“blast exposure”; Gallun et al., 2012, 2016; Saunders et al., 2015; Kubli et al., 2018) and studies of patients with mTBI following non-military events such as falls, sports injuries, and motor vehicle accidents. Even within these two categories, however, there is very little reason to believe that damage to the same brain areas would occur under different physical conditions. Unfortunately, unlike with penetrating head wounds, strokes, or even MS, it is a hallmark of mTBI that only rarely does it result in injuries that can be revealed by current clinical imaging approaches. As reviewed in Gutschalk and Dykstra (2015) and Felix et al. (2018), there is a wide range of ways in which injury to the brain could result in impaired performance on tests of sensitivity to binaural and spatial information. For these reasons, it is essential to interpret the mTBI literature with care and consider carefully the possibility that group differences may not be reliable predictors of what an individual patient may experience.

This heterogeneity across patients may explain why Gallun et al. (2012) found that there was a small, but statistically significant, subset of their injured patients who had abnormally poor MLD scores, but Gallun et al. (2016) were not able to replicate this finding. Instead Gallun et al. (2016) observed data more similar to those of Mueller and Beck (1987), where patients with a history of blast exposure (only some of whom had an mTBI diagnosis) were more likely to show auditory processing difficulties on complex tests but no difficulties on detection tasks such as the MLD. Saunders et al. (2015) also observed abnormal SRM in their larger sample of blast-exposed Veterans, but tested SRM for speech-on-speech masking rather than a detection task. Kubli et al. (2018) explored task complexity explicitly in an SRM task by asking blast-exposed Veterans to localize the voice of a person talking about a specific topic (“sports,” “food,” etc.), either in a quiet room or in the presence of one or more competing speakers talking about other topics. While the injured Veterans performed similarly to a non-blast-exposed control group in quiet, there were significant increases in the group differences in SRM as the complexity of the acoustical environment increased.

Roup and Powell (2016) reported binaural impairment in a group of people who had suffered mTBIs from non-military causes, as did Hoover et al. (2017), who used a wide range of monaural and binaural tests and found significant impairment in the mTBI group. For Hoover et al. (2017), it was impossible to identify a specific monaural or binaural deficit common to all of those with mTBI. These results suggest that both military and non-military brain injury can impair the binaural system, but that knowing the details of the injury, the tests used, and the types of binaural impairment revealed are essential for drawing conclusions that can be used to generalize the results to beyond those patients included in the study.

These results, like the data reviewed from patients who have suffered strokes, developed MS, or are undergoing the normal aging process, reveal the complexity of doing clinical research with patient populations. Nonetheless, shedding light on binaural dysfunction is of great benefit to the patients and to the clinicians who treat them. The binaural system is poorly understood by the general public, and even by most clinical specialists. By clarifying the dysfunction likely to occur among patients with various diseases, it becomes possible to develop new clinical tests as well as to train clinicians in how to counsel those with binaural impairment. In addition, there is potential to deepen our understanding of how the binaural system functions by learning what mechanisms can be impaired and how such mechanisms can change binaural processing.

## Future Directions

The work reviewed in the sections above suggests that, while much has been learned about binaural impairment since the first reports over 100 years ago, there is still much to be discovered. The field would benefit from further research in a number of areas, including animal models and computational modeling. As is clear from this review, the approach of testing participants who have diseases known (or suspected) to affect the binaural system has substantial potential benefit in two different ways. The first is that we are likely to learn more about those diseases and what abilities and difficulties people with those diseases are likely to experience. The second is that these diseases allow scientific investigation of auditory processing that has been perturbed in ways that are otherwise only possible to do in animal research.

It should be noted that despite the many studies and conditions discussed, this review has not been comprehensive, as there has been no discussion of binaural development in children, nor of the effects on binaural function of many additional auditory and brain diseases. In some cases, such as with development, this was due to a need to limit the scope, and in many others it was due to the lack of a well-developed literature. There are many reasons for the limited literature on binaural impairment, both for some of the conditions discussed and for many of those not discussed. The most important is that there are substantial challenges associated with analyzing “nature’s experiments.” The most difficult obstacle is that, unlike in the laboratory, the perturbations of the system are not uniform and are not easily documented. This is why it is of great benefit to develop animal and computational models, where clear relationships can be established between internal modifications of the system and externally measured values. In addition, it is of great value to take advantage of existing human brain imaging technologies and push for the development of new methods that will allow the binaural system to be more clearly revealed.

The other significant obstacle to taking advantage of the binaural impairments imposed by disease, injury, and natural biological processes is one of scientific and clinical culture, rather than techniques or knowledge. While some of the literature cited above reveals collaborations among clinicians and scientists and publications of clinical and basic research in the same journals, much more of it does not. The early literature is striking for a number of reasons, including the creativity and innovation shown in the methods and the insight revealed by the scientists. One aspect that should not be overlooked, however, is the degree to which the work was being done by clinician scientists, testing their own patients using cutting-edge methods. Progress in clinical research on binaural impairment depends on using the newest approaches in testing binaural hearing to better understand the abilities of large numbers of patients with similar disease states, as revealed by the best clinical metrics available. To do this requires us to attend the same conferences, publish in the same journals, and collaborate on grant applications together. Only in this way can new approaches for clinical care be developed and new insight gained into the ways that the binaural system can change its functioning in the course of a human lifetime.

## Author Contributions

The author confirms being the sole contributor of this work and has approved it for publication.

## Conflict of Interest

The author declares that the research was conducted in the absence of any commercial or financial relationships that could be construed as a potential conflict of interest.
